# Zinc allocation to and within *Arabidopsis halleri* seeds: Different strategies of metal homeostasis in accessions under divergent selection pressure

**DOI:** 10.1002/pei3.10032

**Published:** 2020-11-30

**Authors:** Alicja Babst‐Kostecka, Wojciech J. Przybyłowicz, Barbara Seget, Jolanta Mesjasz‐Przybyłowicz

**Affiliations:** ^1^ Department of Environmental Science The University of Arizona Tucson AZ USA; ^2^ Department of Ecology, W. Szafer Institute of Botany Polish Academy of Sciences Krakow Poland; ^3^ Faculty of Physics & Applied Computer Science AGH University of Science and Technology Kraków Poland; ^4^ Department of Botany and Zoology Stellenbosch University Matieland South Africa

**Keywords:** adaptation, facultative metallophyte, homeostasis, metal hyperaccumulation, micro‐PIXE, pseudometallophyte, seed, Zn allocation

## Abstract

- Vegetative tissues of metal(loid)‐hyperaccumulating plants are widely used to study plant metal homeostasis and adaptation to metalliferous soils, but little is known about these mechanisms in their seeds. We explored essential element allocation to *Arabidopsis halleri* seeds, a species that faces a particular trade‐off between meeting nutrient requirements and minimizing toxicity risks.
- Combining advanced elemental mapping (micro‐particle induced X‐ray emission) with chemical analyses of plant and soil material, we investigated natural variation in Zn allocation to *A. halleri* seeds from non‐metalliferous and metalliferous locations. We also assessed the tissue‐level distribution and concentration of other nutrients to identify possible disorders in seed homeostasis.
- Unexpectedly, the highest Zn concentration was found in seeds of a non‐metalliferous lowland location, whereas concentrations were relatively low in all other seed samples—including metallicolous ones. The abundance of other nutrients in seeds was unaffected by metalliferous site conditions.
- Our findings depict contrasting strategies of Zn allocation to *A. halleri* seeds: increased delivery at lowland non‐metalliferous locations (a likely natural selection toward enhanced Zn‐hyperaccumulation in vegetative tissues) versus limited translocation at metalliferous sites where external Zn concentrations are toxic for non‐tolerant plants. Both strategies are worth exploring further to resolve metal homeostasis mechanisms and their effects on seed development and nutrition.

## 1 INTRODUCTION

Exposure to toxic concentration of metals and metalloids, commonly known as heavy metals, remains one of the major environmental health risks around the world. The release of large amounts of metal(loid)s to the soil through either natural geological or anthropogenic processes has adverse effects on physiological processes in plants. This can cause reduced growth rates and even death in sensitive species (Sarma et al., 2018). Some species, however, have adapted to highly contaminated metalliferous soils and can survive and reproduce in these hostile habitats (Baker, 1987; Ernst et al., 2008; Reeves et al., 2017). This tolerance is achieved through the evolution of a complex network of homeostatic mechanisms that regulate metal uptake or exclusion, transport to aerial parts, allocation and detoxification in plant tissues (Memon, 2016). Some of the metals are actually micronutrients (e.g., copper, iron, manganese, molybdenum, nickel, zinc) that—at adequate concentrations—are essential for the normal growth and metabolism of plants. In excessive amounts, however, they may cause toxicity. Plants thus maintain the concentrations of essential metal ions in different cellular compartments within their physiological limits. Such control is particularly interesting in metal‐hyperaccumulating plants, that accumulate extremely high amounts of metal(loid)s in their shoots without developing symptoms of toxicity (Clemens et al., 2002). Possessing the extreme evolutionary traits of metal hyperaccumulation and hypertolerance makes these plants ideally suited for investigations of the regulatory mechanisms that underlie plant metal homeostasis and adaptation to metalliferous environments.

Recent advances in plant genomics, transcriptomics, proteomics and metabolomics have greatly improved our understanding of the physiological, ecological and evolutionary mechanisms involved in hyperaccumulators’ responses to essential metal(oid)s (Babst‐Kostecka et al., 2018; Dar et al., 2018; Dubey et al., 2018; Kajala et al., 2019; Krämer, 2010; Manara et al., 2020; Peng et al., 2020; Sarma et al., 2018; Shanmugam et al., 2013; Verbruggen et al., 2009). These studies were greatly facilitated by the variability in hyperaccumulation and hypertolerance among closely related model species and their populations. For instance, quantitative trait loci (QTL) analyses performed on progenies from the inter‐ and intraspecific crosses between hyperaccumulating, tolerant versus excluder, or non‐tolerant (metal sensitive) plants identified several QTLs for metal accumulation (Deniau et al., 2006; Filatov et al., 2007; Frérot et al., 2010; Willems et al., 2007, 2010). Associations of these QTL regions with hyperaccumulation traits and further transcriptomic and proteomic approaches identified sets of candidate genes and metabolic pathways that regulate metal uptake, transport, distribution and detoxification (Corso et al., 2018; Krämer et al., 2007; van de Mortel et al., 2006; Plessl et al., 2010; Schvartzman et al., 2018; Weber et al., 2004). Overall, these studies have demonstrated that the genetic determinants of metal hyperaccumulation are constitutively overexpressed in hyperaccumulators compared to non‐accumulator species.

While most existing research has focused on the overall growth patterns of plant shoots and roots, relatively little is known about metal homeostasis in seeds. Once nutrients are allocated to the leaves, they need to be further re‐allocated to the seeds in order to complete the plant's life cycle. Concentrations at which elements are supplied to and stored within seeds are critical to initiate the later development of the seedlings and thereby ensure reproductive success. It is generally believed that tolerant plants keep their seeds free from toxic concentrations of metal(loid)s to provide their offspring with a “fresh start” on metalliferous soils (Bothe & Słomka, 2017; Ernst et al., 2000). Accordingly, the metal translocation from maternal to filial tissues, as well as their distribution and concentration within the seeds of hyperaccumulating plants must be strictly controlled to meet the nutrient requirements and at the same time reduce toxicity risk (Eroglu, 2018). It is also expected that the threshold for toxicity is higher in seeds of hyperaccumulators compared to their non‐tolerant relatives. Yet, more research efforts investigating the variation in the elemental distribution in the seeds are needed to gain insight into adaptation and hypertolerance at the seed development stage in hyperaccumulators of essential transition micronutrients that may become toxic in excessive amounts.

Zinc (Zn) is the second most abundant transition metal in living organisms after iron (Fe) (Marschner, 1995). It has important structural, catalytic and activating functions in plants (Lehmann et al., 2014) and in most crops its concentration is below 100 mg/kg dry weight (Pilon‐Smits et al., 2009). Beyond these concentrations, Zn can cause toxicity by replacing other divalent cations involved in the functioning of photosynthetic enzymes (e.g. Fe, magnesium (Mg) and Mn). This replacement of elements interferes with several biochemical, physiological, and structural aspects of plant processes, with adverse effects on photosynthesis, metabolism and plant growth (Dubey et al., 2018; Szopiński et al., 2019; Van Assche & Clijsters, 1986, 1990). In particular, excess Zn has frequently been shown to modify the root system architecture of plants (Dietrich et al., 2019), cause leaf chlorosis associated with the decline of photosystem II efficiency parameters (Balafrej et al., 2020), or induce reactive oxygen species generation (Fernàndez et al., 2012). The first essential pool of Zn in a plant's life cycle is seed Zn, which is mobilized in the growing seedling. Thus, the optimal transport and storage of Zn into seeds is critical for early seedling growth and development (Larkins & Vasil, 2013; Raboy, 1997). Plants differ markedly in their ability to load Zn into seeds and a relatively wide range of Zn concentration in seeds (e.g. 25–100 mg/kg) is considered optimal for early seedling growth (Jones et al., 1991). It has been demonstrated that in cultivated species, the movement of Zn between vegetative tissues and reproductive organs and seeds is affected by the amount of Zn in the former (Longnecker & Robson, 1993). By contrast, the strategy and natural variation in Zn allocation to the seeds of Zn‐hyperaccumulating plants are still little explored.

*Arabidopsis halleri* (L.) O'Kane and Al‐Shehbaz (family Brassicaceae) is an attractive model species to study various aspects of plant adaptation to metalliferous sites, particularly because of its heritable intraspecific variation in Zn hyperaccumulation and hypertolerance capacities (Honjo & Kudoh, 2019). Additionally, *A. halleri* is a pseudometallophyte (facultative metallophyte), that is, a taxon with populations that grow and reproduce on both metalliferous and non‐metalliferous sites (Pollard et al., 2002, 2014). Extreme differences in edaphic conditions between these types of habitats promote rapid differentiation of metallicolous (M) and non‐metallicolous (NM) populations. This local adaptation makes pseudometallophytes particularly relevant for comprehensive, quantitative investigations of the variation in traits involved in plant adaptation to metal‐contaminated soils (Babst‐Kostecka et al., 2014, 2016; Jimenez‐Ambriz et al., 2007; Sailer et al., 2018). Recent advances have placed *A. halleri* at the center of nutrient research in metal hyperaccumulating plants, but existing studies have exclusively addressed vegetative and not reproductive tissues. This could be related to the fact that most studies have been performed in relatively short‐term experiments that involved destructive harvest prior to the seed‐development stage. Still, it is surprising that, to the best of our knowledge, the important elemental concentration and distribution within seeds from natural locations of this intensely studied species have not yet been investigated.

In this study, we investigated the spatial distribution and concentration of elements within the seeds of *A. halleri* populations from non‐metalliferous and metalliferous locations in Southern Poland. We selected four populations, for which earlier investigations of vegetative organs have reported significant quantitative variation in Zn hyperaccumulation and hypertolerance capacities (Babst‐Kostecka et al., 2018; Meyer et al., 2010). While our main focus was on identifying Zn allocation strategies to seeds of non‐metallicolous and metallicolous plants, we also report on the patterns of composition and distribution of other mineral nutrients in these seeds. This is to identify possible interactions between Zn and other elements, as well as disorders in seed homeostasis in the context of adaptation to metalliferous environments. We employed micro‐particle induced X‐ray emission (micro‐PIXE) (Mandò & Przybyłowicz, 2016; Mesjasz‐Przybyłowicz & Przybyłowicz, 2002). Due to the high resolution and sensitivity in performing elemental mapping and quantification of the data extracted from arbitrarily selected micro‐areas of the investigated tissues, this microanalytical method is perfectly suited for trace element analysis of biological samples (Mesjasz‐Przybyłowicz & Przybyłowicz, 2002; Mesjasz‐Przybyłowicz & Przybyłowicz, 2011; Przybyłowicz et al., 1997).

Using this approach, we address the following questions:

1. Do seeds of *A. halleri* plants from anthropogenic and natural habitats differ in elemental distribution and concentration?
2. Is Zn accumulation and allocation inside a seed controlled differently by metallicolous and non‐metallicolous plants?
3. Do metallicolous plants possess a strategy to combat metal stress at the seed level?

In addition, we discuss the emerging patterns of elemental allocation to the seeds in the context of the stark contrast between study sites regarding: differences in hyperaccumulation levels in *A. halleri* leaves, root‐to‐shoot translocation, and soil contamination that we quantified in complementary chemical analyses.

## 2 MATERIALS AND METHODS

### 2.1 Sampling and chemical analyses

**FIGURE 1:**
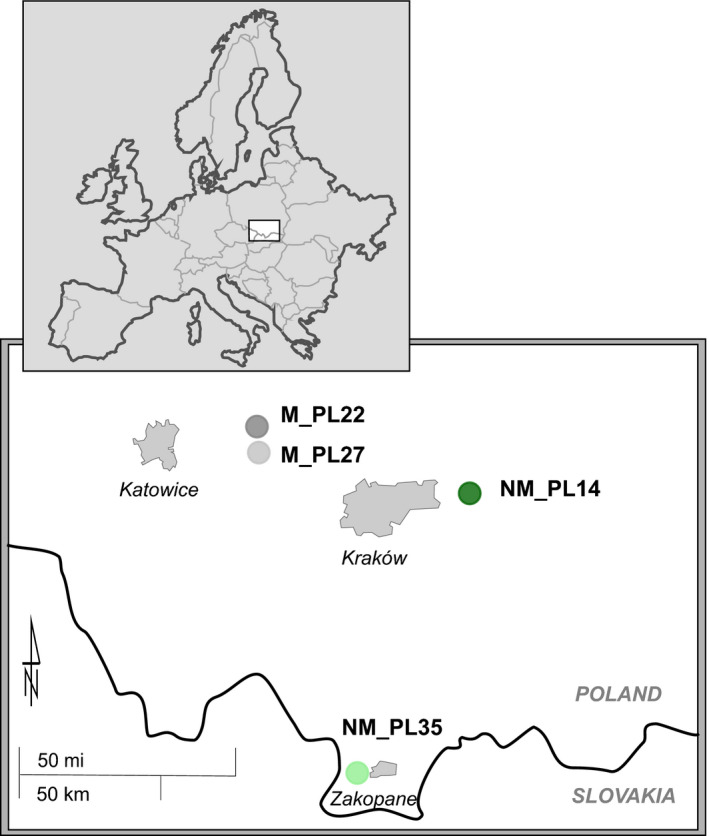
Location of the non‐metalliferous (NM) and metalliferous (M) study sites in southern Poland

Sampling included four *A. halleri* locations in southern Poland at non‐metalliferous (NM) and metalliferous (M) sites (Figure 1, Table 1). The NM_PL14 site is situated in the Sandomierz Basin physiographic macroregion, 20 km east of the city of Kraków, within the northern part of Niepołomice Forest. NM_PL35 grows on the northern foothills of the Western Tatra Mts, near the Kościeliska valley. The metalliferous sites are located in one of the most heavily industrialized regions of Poland (Michalik‐Kucharz, 2008). M_PL22 is located in the vicinity of the Bolesław mine and metallurgical plant near Olkusz, whereas M_PL27 is in the forested area that covers a former open‐pit mine (closed in 1912) near Galman village.

**TABLE 1:** Geographic location, pH, and total concentration of Zn in soil (mean ± *SD*, *n* = 5) at the investigated non‐metalliferous (NM) and metalliferous (M) sites

Edaphic type	Site	Location	Altitude a.s.l. (m)	Latitude	Longitude	Habitat	pH	Zn (mg/kg)
NM	NM_PL14	Niepołomice Forest	188	50°06′31.80″N	20°22′02.88″E	Wet forest edge	5.7 ± 0.5*^c^*	150 ± 40*^c^*
NM	NM_PL35	Western Tatra Mts	927	49°17′13.40"N	19°52′45.90"E	Forest edge	4.5 ± 0.2*^d^*	59 ± 28*^d^*
M	M_PL22	Bukowno	339	50°16′58.08″N	19°28′43.38″E	Woody area	7.2 ± 0.4*^a^*	5,100 ± 3700*^b^*
M	M_PL27	Galman	447	50°11′36.78"N	19°32′15.12"E	Forest	6.1 ± 0.1*^b^*	12,260 ± 5900*^a^*

Note

Different letters indicate statistically significant differences between the four locations at *p ≤ *.05 (Kruskal–Wallis followed by the post hoc test using Fisher's least significant difference criterion).

At each site, five entire *A. halleri* plants together with surrounding soil were sampled every 5 m along transects to avoid clonal repetition. Soil was sampled to a depth of 10 cm using a cylinder of 7 cm diameter and sieved (2 mm mesh). Soil pH was measured in a 1:5 (w: v) suspension of soil in deionized water (ISO 10390). Total nitrogen (N) was measured using the Kjeldahl method and phosphorus (P) using the vanadium–molybdenum method. For the analyses of total Zn, Cd, Pb, Fe, Mg and calcium (Ca) concentrations, dried and ground soil samples were mineralized in suprapur HClO_4_ (1:20 w/v), using a hotplate (FOSS Tecator Digestor Auto). Extracted elements were analyzed with flame or graphite furnace atomic absorption spectrometry (AAS; Varian AA280FS, AA280Z, Agilent Technologies, Santa Clara, USA). Plants were divided into shoots and roots, and washed twice in deionized water. Individual root samples were additionally cleaned in an ultrasonic bath using deionized water (2 min, 30 mHz) and dried at 70°C. To analyze Zn, Cd, Pb in shoots and roots, and Ca, Mg, Fe in shoots, samples were digested in a suprapur mixture of HNO_3_: HClO_4_ (4:1 v/v). Additionally, N and P were quantified in shoots using the Kjeldahl and vanadium–molybdenum methods, respectively. Concentrations of elements in soil and plant extracts were determined with flame or graphite furnace AAS (Varian AA280FS, AA280Z, Agilent Technologies, Santa Clara, USA) and the results ascertained using the certified reference material: CRM048‐050 (RTC) and Standard Reference Material 1570a—Spinach Leaves (National Institute of Standards & Technology), respectively. The recovery values ranged between 95% and 101%, with RDS ±0.5%.

Ripe seeds from ~100 *A. halleri* plants per population were collected as bulk samples. Seeds were air‐dried, which represents their natural physiological dormancy stage. Three seeds from each population were randomly selected for micro‐PIXE analysis.

### 2.2 Elemental distribution and quantification in seeds by micro‐PIXE

Microanalyses of seeds were performed using the nuclear microprobe at the Materials Research Department, iThemba LABS, South Africa. This facility and the methodology of measuring biological materials have been reported earlier in detail (Prozesky et al., 1995; Przybyłowicz et al., 2005; Przybylowicz et al., 1999). Longitudinal sections of the middle parts of dry seeds were mounted on specimen holders between two 0.5% Formvar films. The Formvar film facing proton beam was lightly coated with carbon to prevent charging. A proton beam of 3 MeV energy and a current of 200–300 pA from a 6‐MV single‐ended Van de Graaff accelerator was focused on a 3 × 3 μm^2^ spot and raster scanned over seeds, using rectangular scan patterns with a variable number of pixels (up to 128 x 128). Particle‐induced X‐ray emission (PIXE) and proton elastic backscattering spectrometry (EBS) were used simultaneously. PIXE spectra were registered with a Si(Li) detector (30 mm^2^ active area and 8.5 µm Be window) with an additional 125 µm Be layer as an external absorber. The effective energy resolution of the PIXE system (for the Mn Kα line) was 150–160 eV, measured for individual spectra. The detector was positioned at a takeoff angle of 135 ° and a working distance of 24 mm. The X‐ray energy range was set between 1 and 40 keV. The EBS spectra were recorded with an annular Si surface barrier detector (100 μm thick) positioned at an average angle of 176°. Data were acquired in the event‐by‐event mode. The normalization of results was performed using the integrated beam charge, collected simultaneously from a Faraday cup located behind the specimen and from the insulated specimen holder. The total accumulated charge per scan varied from 1.19 to 6.09 µC.

**FIGURE 2:**
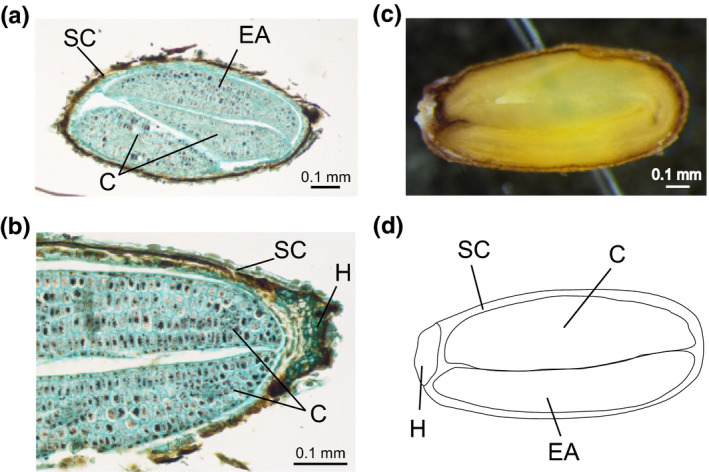
Photomicrograph of a longitudinal section of an *Arabidopsis halleri* seed (a‐c) and schematic representation of regions of interest (ROIs) used in the interpretation of micro‐PIXE results (d). C—cotyledons; SC—seed coat; EA—embryonic axis; H—hilum

Quantitative results were obtained by a standardless method using GeoPIXE II software package (Ryan, 2000; Ryan et al., 1990a). The error estimates were extracted from the error matrix generated in the fit, and the minimum detection limits (MDL) were calculated using the Currie equation (Currie, 1968). Quantitative elemental mapping was performed using Dynamic Analysis method (Ryan, 2000; Ryan & Jamieson, 1993; Ryan et al., 1995). This method generates elemental images, which are (i) overlap‐resolved, (ii) with subtracted background, and (iii) quantitative, that is, accumulated in dry weight units. Maps were complemented by data extracted from arbitrarily selected regions of particular interest (ROIs) within scanned seeds, representing specific seed parts. Five ROIs were selected based on the seed morphological structures (Figure 2). Accordingly, besides the whole seed section, we distinguished the embryo and surrounding seed coat, further detailing from the latter structure the hilum region. Within the embryo, we additionally separated the embryonic axis, cotyledons, and provascular tissues of the entire embryo fraction. Concentrations from these ROIs were obtained from the PIXE spectra fitted using a full nonlinear deconvolution procedure (Ryan et al., 1990a, 1990b). The EBS results confirmed that all seeds met the “infinite thickness” requirement (i.e., absorb all the primary proton beam) and their composition was approximated by the cellulose composition (C_6_H_10_O_5_).

### 2.3 Data analyses

Translocation factors (TF) of Zn, Cd, and Pb in plants were calculated as the ratio of metal concentrations in the shoot and root. As the data did not meet the normality assumption, the differences in the mean concentration of elements between locations were assessed using the multiple comparison Kruskal–Wallis test followed by the post hoc test using the Fisher's least significant difference criterion, when the overall effect was significant. The weighted arithmetic means and their weighted standard deviations of the concentrations of elements quantified using micro‐PIXE were calculated for each population from the three seed replicates using standard formulas (Bevington, 1969; Bevington et al., 1993). As weights we used the inverse of the corresponding standard deviations of the individual measurements. Analyses were performed in R software version 3.3.3 (R Core Team, 2017) using *agricolae* (de Mendiburu, 2014) and *stats* packages.

## 3 RESULTS

### 3.1 General characterization of soil and A. halleri elemental composition

**FIGURE 3:**
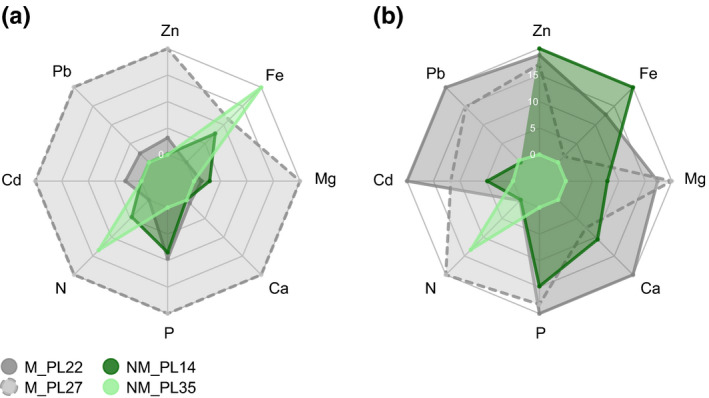
Relative concentrations of elements in soil (a) and *Arabidopsis halleri* shoots (b) at the investigated metalliferous (M) and non‐metalliferous (NM) sites. The outer perimeter indicates the maximum and the central perimeter the minimum value per indicated element

**FIGURE 4:**
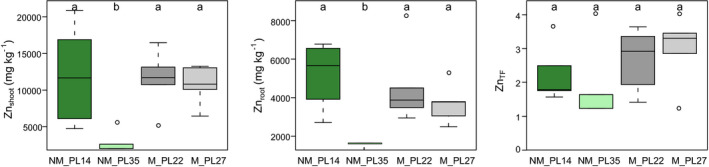
Concentration of Zn in shoots and roots, and translocation factor (TF) in non‐metallicolous (NM) and metallicolous (M) *Arabidopsis halleri* populations. The boxes represent the 25th and 75th percentiles of the data, the whiskers indicate the first and the fourth quartiles, the medians are indicated by the horizontal lines (*n* = 5). The outliers are marked with open dots. Different letters indicate statistically significant differences at *p ≤ *.05

The mean concentration of elements in soil varied broadly between sites. In particular, site M_PL27 showed considerably higher Cd, Pb, and Zn soil concentrations than the M_PL22 site, and very low levels of these elements were found at both NM sites (Figure 3a). The M_PL27 site was also characterized by the highest concentrations of Ca, Mg, N, and P. On the contrary, Fe was most abundant at the NM_PL35 location (Supporting Information Table S1a). Interesting patterns were observed for the nutrients and trace metal elements in *A. halleri* plants (Figure 3b, Supporting Information Table S1b). The most striking was Zn in shoots of the NM_PL14 population, which reached similar concentrations to those found in both M populations, despite considerably lower soil concentration of this element at the NM_PL14 site (Figure 3). Accordingly, only plants from NM_PL35 (except one) did not meet the hyperaccumulation criterion of 3,000 mg/kg of Zn in plant shoots (van der Ent et al., 2013). A similar pattern was found for Cd, with plant shoot concentrations in both M and in NM_PL14 populations exceeding the threshold for Cd hyperaccumulation (100 mg/kg; van der Ent et al., 2013). Again, the NM_PL35 population had no individuals reaching Cd hyperaccumulation. All populations accumulated Zn and Cd mostly in shoots, whereas Pb was mainly accumulated in roots (Figure 4, Supporting Information Figure S1). Plants at the investigated sites did not reach the hyperaccumulation threshold for Pb (1,000 mg/kg; van der Ent et al., 2013). Also, the translocation factor from root to shoot for Pb was below one. Regarding Zn and Cd, the TF > 1 criterion was met in all four populations.

### 3.2 Elemental distribution and quantification in seeds

The micro‐PIXE analysis of *A. halleri* seeds showed the presence of essential macronutrients (P, S, K, Ca), essential micronutrients (Cl, Mn, Fe, Ni, Cu, Zn Mo), potentially beneficial elements (Cr, Rb, Sr, Ti) (Barker & Pilbeam, 2015; Marschner, 1995; Pilon‐Smits et al., 2009), and other elements sporadically appearing in trace amounts (Ga, As, Br and Cd).

In all analyzed seeds, the most significant differences were found for Zn concentration and distribution (Table 2). Surprisingly, seeds from NM_PL14 had the highest average Zn concentration, exceeding the concentrations found in the other NM population (NM_PL35) by a factor of five and those reported for M populations by a factor of three. Furthermore, Zn concentration varied considerably within populations. The broadest range occurred in the M_PL22 population (68–308 mg/kg), whereas M_PL27 was the most homogenous (75–107 mg/kg). It should be noted that these elevated values were still considerably lower than the values observed in shoots and its respective Zn hyperaccumulation threshold of 3,000 mg/kg. Regarding the within‐seed distribution, considerable differentiation in Zn concentration was found for the individual seed structures (ROIs). Hilum represented the region with the highest variation between seeds from the same population. In particular, for the seeds from NM_PL14, the difference between the lowest and the highest concentration of Zn in hilum was only twofold, whereas the respective ratio in seeds from NM_PL35 was 48. By contrast, the most homogeneous Zn concentration was found for the embryonic axis, with a maximum ratio of three within seeds from NM_PL14.

**TABLE 2:** Zinc concentration (micro‐PIXE, mg/kg d.wt,) in the *Arabidopsis halleri* seed cross‐sections

Site	Sample	Whole section	Region of interest (ROI)				
			Embryonic axis	Cotyledon	Provascular strands	Seed coat	Hilum^†^
NM_PL14	1	805 ± 38	461 ± 30	901 ± 44	550 ± 35	1,477 ± 51	7,343 ± 186
	2	393 ± 22	267 ± 21	525 ± 29	NA	222 ± 16	2,975 ± 112
	3	215 ± 14	161 ± 12	139 ± 11	153 ± 12	269 ± 16	3,000 ± 70
	$\bar{X}$	314 ± 10 *^a^*	217 ± 10 *^a^*	225 ± 10*^a^*	190 ± 10*^a^*	300 ± 10*^a^*	3,400 ± 60*^a^*
NM_PL35	1	163 ± 11	110 ± 6	140 ± 11	123 ± 10	169 ± 10	2,347 ± 50
	2	79 ± 6	143 ± 13	65 ± 5	105 ± 9	49 ± 3	394 ± 16
	3	37 ± 4	72 ± 7	32 ± 4	50 ± 7	18 ± 5	49 ± 9
	$\bar{X}$	59 ± 3 *^b^*	99 ± 4 *^b^*	52 ± 3*^b^*	77 ± 4*^b^*	49 ± 2*^b^*	185 ± 8*^a^*
M_PL22	1	308 ± 19	235 ± 17	341 ± 20	264 ± 19	232 ± 13	3,570 ± 77
	2	68 ± 5	116 ± 10	56 ± 5	77 ± 8	57 ± 4	160 ± 13
	3	214 ± 13	141 ± 11	259 ± 15	137 ± 13	109 ± 7	3,659 ± 73
	$\bar{X}$	100 ± 5*^ab^*	144 ± 7*^ab^*	90 ± 5*^ab^*	100 ± 6*^ab^*	81 ± 3*^ab^*	356 ± 13*^a^*
M_PL27	1	96 ± 6	131 ± 8	119 ± 8	106 ± 6	60 ± 7	63 ± 6
	2	75 ± 5	118 ± 9	92 ± 8	119 ± 10	45 ± 3	201 ± 10
	3	107 ± 7	137 ± 12	115 ± 8	126 ± 10	96 ± 11	472 ± 29
	$\bar{X}$	89 ± 3 *^b^*	128 ± 5 *^b^*	109 ± 5*^ab^*	111 ± 4*^ab^*	50 ± 3*^b^*	110 ± 5*^a^*

Note

NA—not available. Weighted means and their weighted *SD* (*n* = 3) are given for every site. Different letters indicate statistically significant differences between the four locations at *p ≤ *.05 (Kruskal–Wallis followed by the post hoc test using Fisher's least significant difference criterion).

^†^ The hilum region was separated from the remaining seed coat section.

**FIGURE 5:**
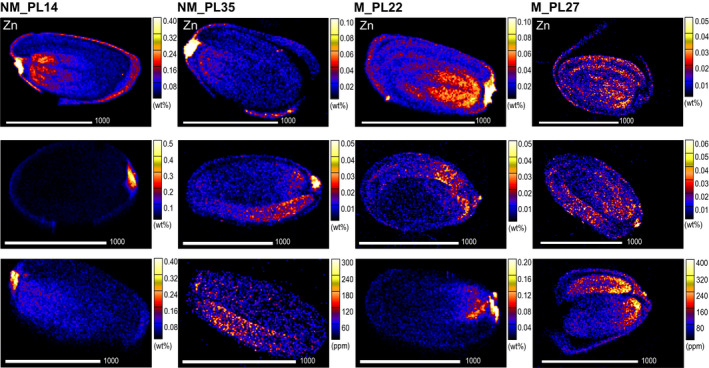
Quantitative Zn concentration (expressed in parts per million, ppm or weight percentage, wt%) maps of *Arabidopsis halleri* seed cross‐sections. Seeds originated from two non‐metallicolous (NM) and two metallicolous (M) populations. Rows represent three randomly selected seeds from each population

Overall, the majority of the analyzed seeds, regardless of plant edaphic origin, accumulated the highest concentrations of Zn in the hilum region (Figure 5). Exceptions were found in single seeds from M_PL27 and NM_PL35, where the highest Zn concentration was found in the embryonic axis. Besides hilum, a considerable Zn concentration was found in the seed coat and in different regions of the embryo. Interestingly, for more than half of the analyzed seeds from both NM and M origins, an increased Zn level was detected in the tip of their cotyledons.

Contrary to the Zn distribution patterns, the ranges of Fe, P, K, Ca, and Mn concentrations were much narrower within and between the studied populations (Table 3, Supporting Information Table S2). Yet, some interesting patterns still emerged. In particular, NM populations had higher P concentration (4500–4600 mg/kg) compared to M populations (3700–4000 mg/kg), whereas the opposite pattern was found for Ca. Furthermore, population NM_PL35 had around twice higher average concentrations of Fe and Mn compared to the remaining populations, and also the highest average K concentration.

**TABLE 3:** Elemental composition (micro‐PIXE, mg/kg d.wt, weighted mean ± weighted *SD*, *n* = 3) of the *Arabidopsis halleri* seed cross‐sections

Element	Site	Whole section	Region of interest (ROI)				
			Embryonic axis	Cotyledon	Provascular strands	Seed coat	Hilum^†^
Fe	NM_PL14	60 ± 1*^b^*	85 ± 3*^ab^*	62 ± 1*^bc^*	120 ± 5*^b^*	16 ± 3*^b^*	312 ± 8*^a^*
	NM_PL35	110 ± 4*^a^*	85 ± 3*^ab^*	77 ± 2*^a^*	170 ± 5*^a^*	34 ± 2*^a^*	264 ± 5*^a^*
	M_PL22	60 ± 2*^b^*	90 ± 3*^b^*	60 ± 2*^c^*	156 ± 5*^b^*	35 ± 1*^a^*	113 ± 2*^ab^*
	M_PL27	50 ± 1*^c^*	35 ± 2*^c^*	71 ± 2*^ab^*	66 ± 3*^b^*	16 ± 1*^b^*	43 ± 3*^b^*
P	NM_PL14	4,600 ± 170*^a^*	1,240 ± 60*^c^*	7,110 ± 240*^a^*	8,515 ± 315*^a^*	130 ± 20*^c^*	4,240 ± 170*^a^*
	NM_PL35	4,560 ± 160*^a^*	6,230 ± 190*^a^*	6,430 ± 210*^b^*	7,210 ± 220*^b^*	620 ± 40*^a^*	1,300 ± 60*^b^*
	M_PL22	4,010 ± 160*^b^*	5,890 ± 180*^ab^*	6,110 ± 190*^c^*	6,660 ± 210*^c^*	610 ± 50*^a^*	1,540 ± 60*^b^*
	M_PL27	3,730 ± 140*^b^*	5,020 ± 180*^b^*	5,560 ± 180*^c^*	6,590 ± 220*^bc^*	490 ± 25*^b^*	445 ± 25*^c^*
K	NM_PL14	2,520 ± 40*^b^*	3,110 ± 45*^b^*	2,940 ± 45*^b^*	3,760 ± 60*^a^*	2,355 ± 20*^b^*	2,280 ± 30*^ab^*
	NM_PL35	3,850 ± 40*^a^*	3,600 ± 50*^a^*	3,340 ± 40*^a^*	3,690 ± 50*^ab^*	3,890 ± 30*^a^*	4,410 ± 20*^a^*
	M_PL22	2,930 ± 40*^ab^*	3,720 ± 50*^a^*	2,980 ± 40*^b^*	3,670 ± 50*^ab^*	3,630 ± 30*^a^*	2,850 ± 30*^ab^*
	M_PL27	2,220 ± 30*^c^*	2,340 ± 30*^c^*	2,270 ± 30*^c^*	2,680 ± 35*^b^*	2,220 ± 20*^b^*	910 ± 10*^b^*
Ca	NM_PL14	2,180 ± 20*^b^*	1,260 ± 15*^a^*	2,460 ± 20*^a^*	1,575 ± 20*^b^*	3,180 ± 16*^b^*	7,350 ± 30*^a^*
	NM_PL35	1,070 ± 13*^c^*	390 ± 7*^b^*	370 ± 7*^b^*	330 ± 7*^c^*	4,470 ± 18*^ab^*	6,740 ± 25*^a^*
	M_PL22	2,360 ± 20*^ab^*	1,350 ± 16*^a^*	2,300 ± 20*^a^*	1,570 ± 20*^b^*	5,230 ± 20*^a^*	5,470 ± 20*^ab^*
	M_PL27	2,380 ± 17*^a^*	1,350 ± 16*^a^*	2,900 ± 20*^a^*	1,970 ± 20*^a^*	3,710 ± 20*^ab^*	3,620 ± 20*^b^*
Mn	NM_PL14	16 ± 1*^c^*	15 ± 1*^b^*	17 ± 1*^bc^*	18 ± 2*^b^*	16 ± 2*^b^*	60 ± 3*^b^*
	NM_PL35	26 ± 1*^a^*	24 ± 2*^a^*	19 ± 1*^ab^*	21 ± 2*^a^*	26 ± 1*^a^*	126 ± 3*^a^*
	M_PL22	14 ± 1*^c^*	16 ± 1*^b^*	11 ± 1*^c^*	14 ± 1*^c^*	15 ± 1*^b^*	47 ± 2*^b^*
	M_PL27	19 ± 1*^b^*	16 ± 1*^b^*	21 ± 1*^a^*	20 ± 1*^a^*	16 ± 1*^b^*	37 ± 2*^c^*

Note

Different letters indicate statistically significant differences between the four locations at *p ≤ *.05 (Kruskal–Wallis followed by the post hoc test using Fisher's least significant difference criterion).

^†^ The hilum region was separated from the remaining seed coat section.

For some seeds, our analyses revealed trace amounts of: Cd (53, 47, and 12 mg/kg in seeds originating from M_PL22 and 9 mg/kg in one seed from M_PL27), Cr (11 mg/kg), Ni (2.5 mg/kg) and Br (1.8 mg/kg)—the latter three elements were detected exclusively in the seeds from NM_PL35. In addition, we found Rb (3 and 10 mg/kg in the seeds from NM_PL14 and M_PL27, respectively), Sr (3 mg/kg in the seeds from populations NM_PL35 and M_PL22), as well as As and Mo (1.7 and 2.1 mg/kg, respectively, in the population M_PL22).

**FIGURE 6:**
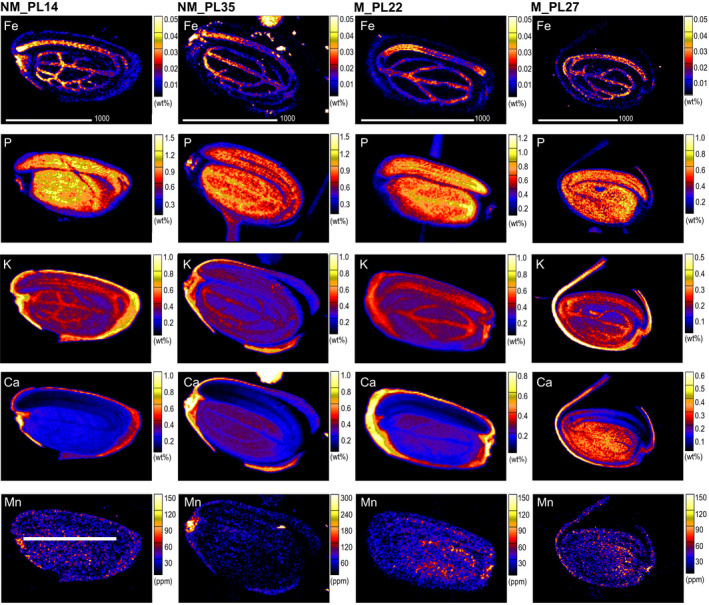
Examples of quantitative elemental maps (parts per million, ppm or weight percentage, wt%) of *Arabidopsis halleri* seed cross‐sections. Seeds originated from two non‐metallicolous (NM) and two metallicolous (M) populations

Regarding the distribution of nutrients within the seeds, similar patterns were observed regardless of plant origin (Figure 6, Table 3, Supporting Information Table S2). Sulfur and P mainly appeared in the embryonic axis and cotyledons; however, the latter element occasionally occurred also in the provascular tissues of those structures. Calcium was mostly present in the hilum and seed coat. Interestingly, in the seeds originating from NM sites, Ca concentrations in the hilum were around twice higher compared to the rest of the seed coat. Calcium concentrations in the cotyledons were about twice lower than in the seed coat, with well visible depletion in the provascular tissue network. The lowest values were observed in the embryonic axis, with the exception of population NM_PL35, where they were comparable in the embryonic axis, cotyledons including the provascular strands, and much lower than for the other populations. The seed coat was also the major region of Cl accumulation. An unambiguous distribution pattern was observed for Fe, which appeared mostly in the embryo parts of the analyzed seeds. For all samples, Fe distribution clearly corresponded with the seed's provascular network. This element was further observed in the hilum of seeds originating from NM sites, but not from M locations. Regarding K, no common pattern among seeds could be found. This element was mostly found in the seed coat and embryo, and in the latter its enrichment further corresponded with the seed's provascular strands distribution. Copper presence was limited exclusively to the seed coat and the hilum of NM samples, whereas in seeds originating from M sites it occurred broadly in all ROIs. For the elements that occurred in trace amounts in some single samples, we could not identify any relevant distribution patterns. Their concentrations were too low (sometimes even below the detection limits of PIXE) to enable elemental mapping.

## 4 DISCUSSION

### 4.1 Exceptionally high Zn accumulation in shoots and seeds of A. halleri from a non‐metalliferous lowland location

This study revealed a broad range of Zn allocation to *A. halleri* seeds and shoots, indicating population‐specific strategies of Zn translocation from the vegetative to filial organs. Surprisingly, the observed patterns were not specific to either ecotype or site‐type and highly contrasting results emerged for the two NM populations. Although the mountain location NM_PL35 was characterized by the lowest Zn concentrations in seeds and shoots, as well as by low root‐to‐shoot translocation, plants from the lowland NM_PL14 site hyperaccumulated four to five times more Zn in all studied organs. Such pronounced differences between NM populations were unexpected, as the amount of Zn in soil was similarly low at both locations.

At non‐contaminated sites, the amount of Zn in vegetative tissues is generally expected to proportionally determine the magnitude of Zn movement to reproductive organs and seeds. This metal homeostasis pattern is well documented in agriculture species (Kranner & Colville, 2011; Longnecker & Robson, 1993) and is likely present in *A. halleri* plants from ancestral non‐metalliferous habitats (e.g. foothills of the Tatra Mts in our study). We thus hypothesize that the exceptionally high internal Zn levels in NM_PL14 plants relate to the remarkable capacity of *A. halleri* to extract Zn from soil at this site. Indeed, a recent study by Dietrich et al. (in review) demonstrated that *A. halleri* accumulated more Zn in shoots at the NM_PL14 location than the majority of accessions studied in field investigations at the European scale, including metalliferous sites (Frérot et al., 2018; Stein et al., 2017). Our findings are further in line with a recent molecular study, where the experimentally assessed quantitative variation in Zn hyperaccumulation between NM *A. halleri* populations was associated with i) the recent colonization of non‐metalliferous lowland locations from M populations, and ii) divergent selection at metalliferous and non‐metalliferous sites that acts toward higher Zn hyperaccumulation levels at non‐metalliferous sites and lower levels at metalliferous sites (Babst‐Kostecka et al., 2018). Indeed, it was concluded that *A. halleri* population NM_PL14 may have actually derived from M populations and that this divergence was associated with evolution toward increased Zn hyperaccumulation capacities at a non‐metalliferous location. Our field investigation now strongly supports the findings of this preceding experimental study.

The increased Zn uptake of *A. halleri* plants at the NM_PL14 site may also be associated with the ability of roots to forage for additional Zn through shifts in root architecture, which is driven by the high internal demand for Zn in this species. A recent rhizobox‐based study demonstrated that NM *A. halleri* produced deeper‐reaching and wider roots when grown in non‐metalliferous compared to metalliferous substrate (Dietrich et al., 2019). The benefits of Zn concentrations in plant tissues when roots explore a greater soil volume has already been shown in short‐term uptake experiments on transgenic plants grown under Zn deficient conditions (Ramesh et al., 2004). This positive trend was associated with an over‐expression of *Arabidopsis* gene AtZIP1, which codes a respective plasma membrane Zn transporter that promotes Zn uptake by the roots. Importantly, the increased abundance of Zn transporters triggered not only enhanced Zn uptake and root‐to‐shoot translocation, but also led to higher Zn content in seeds. In *A. halleri,* members of ZIP gene family have very high expression levels, which is linked with Zn hypertolerance and hyperaccumulation abilities of this species (Hanikenne et al., 2008, 2013). Also, more recent work performed on two populations from M sites in Italy and Poland indicated that in M *A. halleri* populations, Zn accumulation in shoots is mostly determined by root processes (Schvartzman et al., 2018).

Our study demonstrated that selection toward enhanced Zn accumulation capabilities in vegetative tissues at lowland NM locations increases the delivery of Zn from the mother plant to its seeds. The highest Zn concentrations of the whole‐seed cross‐sections were found in the NM_PL14 population. Seeds from this location contained three times more Zn than the upper limit of seed storage that is considered optimal for early seedling growth and development (Raboy, 1997). By contrast, all other investigated populations developed seeds with Zn concentrations falling within this critical range. Furthermore, the relatively low Zn concentrations in seeds of both M populations indicated that *A. halleri* plants growing at metalliferous sites carefully regulate the uptake, distribution and concentration of metals in their seeds. Restriction of Zn allocation to seeds at metalliferous sites has also been reported for the Zn hyperaccumulator *Thlaspi praecox* (Vogel‐Mikus et al., 2007), as well as for the Zn excluders *Biscutella laevigata* (Babst‐Kostecka et al., 2020; Mesjasz‐Przybyłowicz et al., 2001), *Gypsophila fastigiata* and *Silene vulgaris* (Mesjasz‐Przybyłowicz et al., 1998, 1999). This well‐known tolerance strategy prevents excessive levels of metal(loid)s in seeds to minimize negative effects on seed germination (Bothe & Słomka, 2017; Ernst et al., 1992; Mesjasz‐Przybyłowicz et al., 2001). Accordingly, plants growing on metal‐contaminated soils usually develop seeds that contain lower concentrations of metals than their vegetative parts. This regulation and distribution pattern is particularly important in hyperaccumulators, which are known for their very effective root‐to‐shoot metal(loid) translocation mechanisms through symplastic movement and xylem loading (Eroglu, 2018; Verbruggen et al., 2009).

### 4.2 The importance of hilum for the regulation of Zn transport to A. halleri seeds

Given the broad variability in Zn accumulation at the whole‐seed cross‐section level, the strategy of Zn allocation within NM and M seeds is particularly interesting and relevant to better understand metal regulation and homeostasis mechanisms, as well as their role in metal tolerance. Hilum was a primary region of Zn accumulation in almost all seeds, but the amount of Zn in the hilum relative to other seed regions differed between populations. The pattern of metal(loid)s accumulating mainly in the hilum region has previously been reported for seeds of *G. fastigiata* and *A. thaliana* metal‐accumulating mutant (Mesjasz‐Przybyłowicz et al., 1998; Young et al., 2007), suggesting an important role of the hilum in the formation of seeds in plants. Indeed, hilum represents the first point of entry for nutrients destined for filial seed compartments via the funiculus. This is further consistent with the finding that the mother plant has the ability to control the amount of Zn inside a seed (Olsen et al., 2016). Using *A. thaliana* mutants, the authors demonstrated that heavy metal‐transporting P1B‐ATPases (HMAs) are required for the successful export of Zn from the maternal to the filial parts of the seeds, and that this translocation is blocked without the combined function of two Zn transporters (AtHMA2 and AtHMA4) in the seed coat. Importantly, HMA4 is also an important regulator of Zn hyperaccumulation in *A. halleri* (Nouet et al., 2015) and it has been reported to be triplicated in this species (Hanikenne et al., 2008, 2013). Our results from *A. halleri* accessions growing at NM and M field locations provide further support for the relevance of the hilum region of the seed coat and for maternal regulation of Zn translocation to the seeds. By preserving the Zn surplus in the hilum region, *A. halleri* plants likely prevent its accumulation across entire seeds at harmful levels, which could hamper successful reproduction. Thus, our study suggests that functional Zn pumps in the seed coat are essential, not only to enable Zn translocation across biological membranes and into filial tissues, but also to prevent excessive Zn accumulation across the entire seed.

Outside the hilum region Zn appeared to be evenly distributed across the *A. halleri* seed. The relatively high and proportional allocation to the seed coat is likely due to the inclusion of the endosperm within this region in our study. In contrast to the outermost protective layer of the seed that has often been reported to contain the lowest amount of Zn within the seed (Babst‐Kostecka et al., 2020; Vogel‐Mikus et al., 2007), the endosperm is a nutrient storage organ rich in Zn. Yet, the endosperm in *Arabidopsis* seeds consists of a very thin single‐cell layer, which blends with the seed coat in image analyses of elemental distribution in such small‐sized seeds. The relatively widespread and uniform presence of Zn in the remaining parts of *A. halleri* seeds is not surprising, as this element is utilized in protein synthesis, membrane structure and functions, gene expression and oxidative stress tolerance (Cakmak, 2000; Robson, 2012). Thus, Zn is needed in metabolically active differentiating cells that will form root and shoot tissues in the developing seedling. High metal(loid) concentrations in seeds are also critically important for the protection of germinating seeds and developing seedlings against infection by soil‐borne and seed‐borne pathogens, especially under environmentally stressful conditions (Carvalho et al., 2020; Carvalho et al., 2020; Ozturk et al., 2006).

### 4.3 Abundance of essential elements (other than Zn) in A. halleri seeds is unaffected by metalliferous site conditions

Our study demonstrated that the bio‐mineralization processes during seed development are efficient in both NM and M ecotypes of *A. halleri*. The quantification of macro‐ and micronutrients indicated an optimal mineral nutrition and standard distribution patterns of elements within the seed. Overall, these elements were present in all samples and their allocation to different structures was clearly element specific and not ecotype specific. Thus, regarding essential nutrients, *A. halleri* seed composition did not depend on metalliferous or non‐metalliferous environment. This is in agreement with previous work performed under hydroponic conditions, where Zn treatment had very limited impacts on (micro)nutrient homeostasis in M *A. halleri* populations (Schvartzman et al., 2018).

Patterns of K and Ca distribution between seed tissues of *A. halleri* followed the general trends in other plants and represented abundant storage of these elements in seeds in preparation for germination (Lott & West, 2001). Potassium and Ca are often associated with the storage of P, as phytate—the main form of P in in the seed—contains K and Ca (White & Veneklaas, 2012). Phytate deposits are evenly distributed throughout the tissues of cotyledons, which is in accordance with the P allocation pattern that we observed in our study. Importantly, the negatively charged P in phytates strongly binds to metal cations such as Zn^2+^ (Bohn et al., 2008). When metal ions are in excess, insoluble mixed salts form, that make both elements unavailable as nutritional factors (Torres et al., 2005). Our study suggests a relationship between the homeostasis of these two nutrients in *A. halleri* seeds. In particular, extremely high concentrations of both P and Zn in the hilum of NM_PL14 seeds might represent the formation of insoluble P–Zn complexes under mechanisms of plant control of P–Zn subcellular location and intracellular transport. Indeed, transcriptomics studies and experiments on *A. thaliana* mutants revealed the genetic background of Zn and P homeostasis co‐regulation and highlighted its involvement in the adaptive responses to P/Zn deficiency/excess stresses in plants (Huang et al., 2000; Khan et al., 2014).

Overall, these results indicate that regarding the regulation of nutrient uptake and distribution within the seed, *A. halleri* plants are well adapted to metalliferous environments. Developing high‐quality seeds is essential to colonize and reproduce in hostile habitats.

## 5 CONCLUSIONS AND PERSPECTIVES

In this study, we visualized and quantified a range of elemental allocation patterns within the seeds of the Zn/Cd‐hyperaccumulating pseudometallophyte *A. halleri* from metalliferous and non‐metalliferous sites. The patterns that we observed—especially the quantitative variation in Zn accumulation and the fact that the abundance and distribution of other nutrients within seeds were unaffected by metalliferous site conditions—indicate the adaptation to metalliferous environments at the seed developmental stage. Importantly, the strategies of Zn allocation within NM and M seeds from lowland locations differ and likely depend on the opposite direction of natural selection in metal‐contaminated and uncontaminated habitats. Overall, this calls for interdisciplinary studies that address the evolutionary history and quantitative variability in Zn accumulation, as well as the impacts of the plant internal and external environment on seed morphology, germination and seedling establishment. Such investigations are needed to identify, which seed characteristics represent local adaptation and which are associated with a cost of such adaptation to the selective environments at metalliferous and non‐metalliferous locations. The distinction between benefic and malefic traits is essential to deepen our understanding of metal homeostasis mechanisms, as well as their ecological and evolutionary effects on the development and nutrition of seeds.

## CONFLICT OF INTEREST

The authors declare no conflict of interest.

[Correction added on 11 June 2021, after first online publication: Conflict of Interest statement added to provide full transparency.]

## AUTHOR CONTRIBUTIONS

JMP planned and designed the research with critical input from ABK. BS prepared seeds for analysis. WJP performed the micro‐PIXE measurements. WJP, JMP and BS processed the image analyses. ABK, JM‐P and WJP analyzed the data. ABK wrote the manuscript with contributions from all authors.

## Supporting information

Supplementary Material
