# Supplementary material for: Zinc allocation to and within Arabidopsis halleri seeds: Different strategies of metal homeostasis in accessions under divergent selection pressure
Source: Plant Environ Interact. 2020 Nov 30;1(3):207–20. doi: 10.1002/pei3.10032 (PMC10168052; doi:10.1002/pei3.10032)
Supplement: Supplementary file 1 — Supplementary Material [file PEI3-1-207-s001.docx]

## Supporting Information

Article title: **Zinc allocation to and within *Arabidopsis halleri* seeds: Different strategies of metal homeostasis in accessions under divergent selection pressure**

Authors: Alicja Babst-Kostecka, Wojciech Przybyłowicz, Barbara Seget, Jolanta Mesjasz-Przybyłowicz

The following Supporting Information is available for this article:

**Table S1** Total concentration of elements in soil (**a**) and *Arabidopsis halleri* shoots (**b**) at the investigated metalliferous (M) and non-metalliferous (NM) sites (n=5). Different letters indicate statistically significant differences between the four locations at *P* ≤ 0.05.

**Table S2** Elemental composition (micro-PIXE, mg kg^-1^ d.wt) of the *Arabidopsis halleri* seed cross-sections. The results are obtained from individual micro-PIXE maps, based on the PIXE spectra fitted using a full nonlinear deconvolution procedure, with analytical errors of each analysis. Stars mark the results obtained from maps of elements shown in Figure 6. Note that the hilum region was separated from the remaining seed coat section. NA – data not available.

**Figure S1** Concentration of Cd and Pb in shoots and roots, and translocation factor (TF) in non-metallicolous (NM) and metallicolous (M) *Arabidopsis halleri* populations. The box represents the 25^th^ and 75^th^ percentiles of the data, the whiskers indicate the first and the fourth quartiles, the median is indicated by the horizontal line. Different letters indicate statistically significant differences at P ≤ 0.05.

**Table S1** Total concentration (mg kg^-1^**)** of elements in soil (**a**) and *Arabidopsis halleri* shoots (**b**) at the investigated metalliferous (M) and non-metalliferous (NM) sites. Different letters indicate statistically significant differences between the four locations at *P* ≤ 0.05.

**a)**

| **Site** | **Sample nb** | **Zn** | **Cd** | **Pb** | **N** | **P** | **Mg** | **Ca** | **Fe** |
| --- | --- | --- | --- | --- | --- | --- | --- | --- | --- |
| NM_PL14 | 1 | 176 | 0.45 | 28 | 3210 | 710 | 6900 | 3200 | 15940 |
| NM_PL14 | 2 | 190 | 0.71 | 28 | 2730 | 630 | 6390 | 2840 | 14860 |
| NM_PL14 | 3 | 87 | 0.19 | 17 | 2820 | 710 | 7510 | 3590 | 17150 |
| NM_PL14 | 4 | 143 | 0.40 | 20 | 3070 | 820 | 9310 | 9100 | 19400 |
| NM_PL14 | 5 | 152 | 0.32 | 26 | 3250 | 810 | 9440 | 9800 | 17540 |
|  | *mean ± SD* | *150 ± 40 ^c^* | *0.42 ± 0.19 ^c^* | *24 ± 5 ^d^* | *3016 ± 232 ^b^* | *736 ± 79 ^b^* | *7910 ± 1396 ^b^* | *5706 ± 3437 ^b^* | *16978 ± 1716 ^a^* |
| NM_PL35 | 1 | 29 | 0.20 | 27 | 4320 | 350 | 3789 | 9785 | 38772 |
| NM_PL35 | 2 | 81 | 0.11 | 30 | 5672 | 320 | 2214 | 4060 | 10041 |
| NM_PL35 | 3 | 74 | 0.21 | 31 | 5660 | 323 | 2942 | 2041 | 22014 |
| NM_PL35 | 4 | 29 | 0.20 | 27 | 5846 | 292 | 2650 | 4145 | 14458 |
| NM_PL35 | 5 | 81 | 0.11 | 30 | 4398 | 350 | 3872 | 9586 | 37990 |
|  | *mean ± SD* | *59 ± 28 ^d^* | *0.17 ± 0.05^d^* | *29 ± 2 ^c^* | *5179 ± 753 ^a^* | *327 ± 24 ^c^* | *3093 ± 722 ^d^* | *5924 ± 3537 ^b^* | *24655 ± 13244 ^a^* |
| M_PL22 | 1 | 3350 | 31 | 1165 | 1400 | 690 | 3900 | 6290 | 11660 |
| M_PL22 | 2 | 3886 | 24 | 945 | 2240 | 790 | 3770 | 5450 | 11170 |
| M_PL22 | 3 | 3151 | 22 | 934 | 1350 | 790 | 7050 | 9770 | 12390 |
| M_PL22 | 4 | 11744 | 88 | 2217 | 2720 | 870 | 5440 | 9100 | 11950 |
| M_PL22 | 5 | 3310 | 26 | 919 | 1760 | 820 | 6920 | 10220 | 13700 |
|  | *mean ± SD* | *5088 ± 3731^b^* | *38 ± 28 ^b^* | *1236 ± 558 ^b^* | *1894 ± 583 ^c^* | *792 ± 66 ^b^* | *5416 ± 1576 ^c^* | *8166 ± 2154 ^b^* | *12174 ± 961 ^b^* |
| M_PL27 | 1 | 21912 | 117 | 8386 | 3530 | 1100 | 50030 | 88190 | 19040 |
| M_PL27 | 2 | 11177 | 70 | 8083 | 6540 | 1300 | 41590 | 58020 | 21610 |
| M_PL27 | 3 | 6284 | 87 | 1544 | 9590 | 1450 | 14840 | 33320 | 14230 |
| M_PL27 | 4 | 12602 | 130 | 9079 | 5200 | 1180 | 47970 | 74630 | 23520 |
| M_PL27 | 5 | 9316 | 90 | 2433 | 9110 | 1410 | 25240 | 49220 | 17960 |
|  | *mean ± SD* | *12258 ± 5891 ^a^* | *99 ± 24 ^a^* | *5905 ± 3607 ^a^* | *6794 ± 2571 ^a^* | *1288 ± 149 ^a^* | *35934 ± 15288 ^a^* | *60676 ± 21438 ^a^* | *19272 ± 3561* |

**b)**

| **Site** | **Sample nb** | **Zn** | **Cd** | **Pb** | **N** | **P** | **Mg** | **Ca** | **Fe** |
| --- | --- | --- | --- | --- | --- | --- | --- | --- | --- |
| NM_PL14 | 1 | 16882 | 166 | 0.1 | 21117 | 4430 | 5365 | 14949 | 1014 |
| NM_PL14 | 2 | 20883 | 208 | 0.4 | 18576 | 1785 | 5315 | 22082 | 60 |
| NM_PL14 | 3 | 11668 | 45 | 0.8 | 26379 | 2696 | 2286 | 12132 | 344 |
| NM_PL14 | 4 | 4738 | 25 | 1.0 | 15867 | 3588 | 2899 | 24225 | 636 |
| NM_PL14 | 5 | 6109 | 92 | 0.9 | 28041 | 4091 | 3019 | 16303 | 473 |
|  | *mean ± SD* | *12056 ± 6897 ^a^* | *107 ± 78 ^b^* | *0.6 ± 0.4 ^b^* | *21996 ± 5143 ^c^* | *3318 ± 1078 ^ab^* | *3777 ± 1454 ^b^* | *17938 ± 5050 ^ab^* | *505 ± 354 ^a^* |
| NM_PL35 | 1 | 2617 | 25 | 0.3 | 29830 | 2412 | 1542 | 5939 | 67 |
| NM_PL35 | 2 | 2013 | 19 | 2.6 | 31484 | 2736 | 1593 | 5143 | 109 |
| NM_PL35 | 3 | 5683 | 23 | 2.0 | 31134 | 2200 | 2717 | 8385 | 190 |
| NM_PL35 | 4 | 2617 | 25 | 0.3 | 34100 | 2412 | 1542 | 5940 | 67 |
| NM_PL35 | 5 | 2013 | 19 | 2.6 | 35400 | 2736 | 1593 | 5144 | 109 |
|  | *mean ± SD* | *2989 ± 1536 ^b^* | *22 ± 3 ^c^* | *1.5 ± 1.2 ^b^* | *32390 ± 2288 ^b^* | *2499 ± 233 ^b^* | *1798 ± 515 ^c^* | *6110 ± 1332 ^c^* | *108 ± 50 ^b^* |
| M_PL22 | 1 | 16473 | 395 | 49 | 21300 | 4172 | 7602 | 56129 | 327 |
| M_PL22 | 2 | 13139 | 345 | 52 | 23400 | 4059 | 6962 | 30371 | 565 |
| M_PL22 | 3 | 10738 | 329 | 21 | 24200 | 3192 | 6295 | 20503 | 314 |
| M_PL22 | 4 | 11702 | 583 | 29 | 20800 | 3103 | 5688 | 19970 | 260 |
| M_PL22 | 5 | 5445 | 216 | 28 | 21090 | 3478 | 4296 | 15597 | 339 |
|  | *mean ± SD* | *11500 ± 4022 ^a^* | *373 ± 134 ^a^* | *36 ± 14 ^a^* | *22158 ± 1536 ^c^* | *3601 ± 491 ^a^* | *6169 ± 1269 ^a^* | *28514 ± 16356 ^a^* | *361 ± 118 ^a^* |
| M_PL27 | 1 | 10813 | 83 | 30 | 37600 | 4995 | 6808 | 15740 | 145 |
| M_PL27 | 2 | 6457 | 123 | 26 | 37583 | 2194 | 3692 | 11038 | 45 |
| M_PL27 | 3 | 13254 | 95 | 12 | 35753 | 3173 | 7858 | 13441 | 140 |
| M_PL27 | 4 | 10096 | 439 | 18 | 39412 | 3796 | 7489 | 17414 | 157 |
| M_PL27 | 5 | 13044 | 402 | 47 | 37600 | 3369 | 8379 | 14696 | 211 |
|  | *mean ± SD* | *10733 ± 2757 ^a^* | *228 ± 176 ^ab^* | *27 ± 13 ^a^* | *37590 ± 1294 ^a^* | *3505 ± 1019 ^ab^* | *6845 ± 1853 ^a^* | *14466 ± 2407 ^b^* | *140 ± 60 ^b^* |

**Table S2** Elemental composition (micro-PIXE, mg kg^-1^ d.wt) of the *Arabidopsis halleri* seed cross-sections. The results are obtained from individual micro-PIXE maps, based on the PIXE spectra fitted using a full nonlinear deconvolution procedure, with analytical errors of each analysis. Stars mark the results obtained from maps of elements shown in Figure 6. Note that the hilum region was separated from the remaining seed coat section. NA – data not available.

| **Element** | **Sample** | **Region of interest (ROI)** | | | | | |
| --- | --- | --- | --- | --- | --- | --- | --- |
|  |  | **Whole section** | **Embrionic axis** | **Cotyledon** | **Provascular tissues** | **Seed coat** | **Hilum** |
| P | NM_PL14_1* | 5467±344 | 7434±385 | 8246±477 | 9681±513 | 33±34 | 4706±362 |
|  | NM_PL14_2 | 4077±253 | 932±62 | 6461±382 | NA | 137±26 | 5087±320 |
|  | NM_PL14_3 | 4667±282 | 5896±345 | 7040±375 | 7789±399 | 851±101 | 3551±240 |
|  | NM_PL35_1* | 4473±276 | 5546±318 | 7026±398 | 7526±403 | 691±70 | 5365±417 |
|  | NM_PL35_2 | 5387±335 | 6712±361 | 7058±387 | 8198±433 | 389±69 | 930±68 |
|  | NM_PL35_3 | 4237±224 | 6521±316 | 5716±300 | 6520±341 | 773±69 | 2044±113 |
|  | M_PL22_1* | 3902±275 | 6092±271 | 6263±313 | 7145±422 | 663±77 | 2154±171 |
|  | M_PL22_2 | 4115±275 | 5451±339 | 5681±336 | 6513±346 | 587±91 | 1337±67 |
|  | M_PL22_3 | 4021±264 | 5982±311 | 6311±301 | 6541±351 | 568±72 | 2615±216 |
|  | M_PL27_1* | 3294±206 | 4527±263 | 4925±255 | 5664±326 | 487±48 | 496±42 |
|  | M_PL27_2 | 3680±226 | 4631±280 | 6296±378 | 6939±408 | 470±49 | 324±32 |
|  | M_PL27_3 | 4561±274 | 6893±393 | 6130±335 | 7813±426 | 494±38 | 1600±114 |
| K | NM_PL14_1* | 3791±94 | 3582±92 | 3409±101 | 3905±109 | 6883±48 | 7300±89 |
|  | NM_PL14_2 | 2001±58 | 2515±73 | 2479±73 | NA | 1043±37 | 1757±85 |
|  | NM_PL14_3 | 2558±59 | 3349±66 | 3131±70 | 3695±76 | 1388±31 | 1589±35 |
|  | NM_PL35_1* | 2773±68 | 2843±85 | 2911±77 | 3196±80 | 3604±49 | 5457±63 |
|  | NM_PL35_2 | 3164±81 | 3596±92 | 3589±87 | 3931±95 | 2408±44 | 1346±29 |
|  | NM_PL35_3 | 5022±59 | 4079±68 | 3507±64 | 3992±71 | 13656±105 | 16582±60 |
|  | M_PL22_1* | 3098±61 | 3612±83 | 3377±82 | 3888±83 | 3711±48 | 3775±36 |
|  | M_PL22_2 | 2793±65 | 3927±84 | 2639±64 | 3699±79 | 2950±53 | 1979±56 |
|  | M_PL22_3 | 2886±67 | 3595±93 | 3115±75 | 3467±80 | 4657±71 | 1671±52 |
|  | M_PL27_1* | 1952±42 | 1784±46 | 1755±45 | 2049±51 | 3368±42 | 2518±104 |
|  | M_PL27_2 | 2291±51 | 2840±65 | 3143±66 | 3654±78 | 1481±31 | 748±13 |
|  | M_PL27_3 | 2587±55 | 2960±65 | 2457±61 | 2996±64 | 2969±78 | 2824±49 |
| Fe | NM_PL14_1* | 79±3 | 97±5 | 99±3 | 239±12 | 41±7 | 305±14 |
|  | NM_PL14_2 | 52±2 | 111±6 | 49±2 | NA | 11±3 | 130±25 |
|  | NM_PL14_3 | 57±3 | 66±4 | 54±3 | 94±5 | 41±2 | 344±10 |
|  | NM_PL35_1* | 148±7 | 422±20 | 69±3 | 200±9 | 33±2 | 221±8 |
|  | NM_PL35_2 | 116±6 | 72±3 | 89±5 | 166±9 | 38±3 | 376±8 |
|  | NM_PL35_3 | 78±6 | 129±9 | 93±7 | 263±15 | 30±4 | 56±14 |
|  | M_PL22_1* | 55±3 | 86±4 | 71±3 | 180±10 | 38±2 | 36±5 |
|  | M_PL22_2 | 56±3 | 108±7 | 44±3 | 159±10 | 27±4 | 423±12 |
|  | M_PL22_3 | 83±4 | 91±6 | 62±3 | 139±10 | 33±2 | 836±28 |
|  | M_PL27_1* | 54±3 | 93±5 | 64±3 | 175±10 | 15±1 | 34±3 |
|  | M_PL27_2 | 43±2 | 49±3 | 60±3 | 149±8 | 16±1 | 68±5 |
|  | M_PL27_3 | 66±3 | 19±2 | 124±5 | 37±4 | 29±5 | 35±12 |
| S | NM_PL14_1 | 12088±140 | 15470±89 | 15266±104 | 13973±76 | 2060±101 | 5682±233 |
|  | NM_PL14_2 | 8907±114 | 10669±98 | 11130±108 | NA | 1017±35 | 2388±80 |
|  | NM_PL14_3 | 10366±115 | 14229±124 | 11508±122 | 12381±132 | 4590±107 | 2078±93 |
|  | NM_PL35_1 | 9042±95 | 12356±88 | 11465±122 | 10333±94 | 4465±126 | 2524±124 |
|  | NM_PL35_2 | 12698±137 | 17106±99 | 15325±123 | 15346±111 | 1588±62 | 1443±60 |
|  | NM_PL35_3 | 10147±77 | 13343±61 | 11541±65 | 11575±52 | 6425±99 | 3306±47 |
|  | M_PL22_1 | 6823±125 | 10186±67 | 8676±68 | 8169±91 | 3381±123 | 1739±79 |
|  | M_PL22_2 | 9444±148 | 13796±143 | 9878±105 | 11397±119 | 4709±123 | 4018±133 |
|  | M_PL22_3 | 9094±120 | 13838±116 | 10374±82 | 11247±115 | 3685±115 | 2376±107 |
|  | M_PL27_1 | 5253±76 | 6621±57 | 5282±62 | 5352±44 | 4392±120 | 2695±67 |
|  | M_PL27_2 | 6699±99 | 10340±123 | 8048±102 | 8487±96 | 3481±92 | 687±30 |
|  | M_PL27_3 | 8540±119 | 10838±96 | 10040±116 | 8560±111 | 3853±88 | 2305±95 |
| Ca | NM_PL14_1* | 1843±29 | 980±22 | 1800±32 | 1212±27 | 3966±32 | 6546±33 |
|  | NM_PL14_2 | 2425±32 | 1577±29 | 3133±38 | NA | 2133±28 | 5960±80 |
|  | NM_PL14_3 | 2349±31 | 1450±32 | 2737±39 | 2090±32 | 3554±26 | 10118±54 |
|  | NM_PL35_1* | 2281±30 | 1113±23 | 2746±46 | 1748±32 | 3107±27 | 9091±34 |
|  | NM_PL35_2 | 2493±42 | 1339±26 | 2100±40 | 1636±34 | 6531±27 | 4420±42 |
|  | NM_PL35_3 | 524±16 | 214±8 | 262±7 | 208±7 | 1579±55 | 3535±67 |
|  | M_PL22_1* | 2272±35 | 1098±24 | 2012±32 | 1288±27 | 5133±34 | 5700±18 |
|  | M_PL22_2 | 2520±48 | 1403±32 | 2198±44 | 1714±36 | 5516±35 | 5485±51 |
|  | M_PL22_3 | 2377±37 | 1625±31 | 2766±37 | 1947±36 | 4804±52 | 3776±48 |
|  | M_PL27_1* | 1921±25 | 1113±22 | 2645±25 | 1777±24 | 2467±44 | 1810±32 |
|  | M_PL27_2 | 2648±31 | 1390±28 | 3232±45 | 2089±36 | 3316±27 | 4087±38 |
|  | M_PL27_3 | 3090±40 | 1965±44 | 3317±41 | 2634±50 | 4765±30 | 5912±40 |
| Cl | NM_PL14_1 | 84±25 | 34±27 | 8±23 | <35 | 282±18 | 313±49 |
|  | NM_PL14_2 | 38±19 | 27±14 | 52±22 | NA | 24±13 | <37 |
|  | NM_PL14_3 | 121±32 | 138±38 | 123±31 | 195±36 | 158±14 | 57±20 |
|  | NM_PL35_1 | 22±19 | 50±24 | <17 | <21 | 95±11 | <21 |
|  | NM_PL35_2 | 54±19 | <24 | 31±24 | 23±23 | 194±13 | 84±7 |
|  | NM_PL35_3 | 65±27 | 61±31 | 43±31 | 63±30 | 115±22 | 50±19 |
|  | M_PL22_1 | 58±19 | 41±23 | 48±22 | 34±23 | 127±18 | 67±15 |
|  | M_PL22_2 | 20±14 | <18 | 12±18 | <21 | 61±8 | 56±24 |
|  | M_PL22_3 | 23±14 | <18 | 13±20 | 20±15 | 55±13 | <28 |
|  | M_PL27_1 | 126±17 | 71±14 | 115±17 | 84.5±20 | 235±12 | 358±21 |
|  | M_PL27_2 | 18±12 | <15 | <17 | <19 | 43±6 | 18±11 |
|  | M_PL27_3 | 99±20 | 85±24 | 107±20 | 77±31 | 164±18 | 102±25 |
| Mn | NM_PL14_1* | 13±1 | 10±2 | 15±1 | 11±3 | 21±5 | 33±5 |
|  | NM_PL14_2 | 17±1 | 13±2 | 22±1 | NA | 13±3 | 45±7 |
|  | NM_PL14_3 | 17±1 | 22±2 | 16±1 | 22±2 | 17±2 | 94±5 |
|  | NM_PL35_1* | 19±2 | 17±3 | 14±1 | 13±3 | 23±2 | 154±7 |
|  | NM_PL35_2 | 31±2 | 25±2 | 31±2 | 29±3 | 39±3 | 126±5 |
|  | NM_PL35_3 | 28±3 | 35±5 | 41±4 | 29±4 | 19±3 | 107±6 |
|  | M_PL22_1* | 21±1 | 22±2 | 29±2 | 26±3 | 14±2 | 43±3 |
|  | M_PL22_2 | 9±1 | 16±2 | 6±0.9 | 12±3 | 14±4 | 46±5 |
|  | M_PL22_3 | 12±1 | 9±2 | 12±0.8 | 10±2 | 16±2 | 74±7 |
|  | M_PL27_1* | 17±1 | 16±1 | 19±1 | 19±2 | 17±2 | 29±3 |
|  | M_PL27_2 | 18±1 | 13±1 | 22±1 | 23±3 | 10±2 | 36±3 |
|  | M_PL27_3 | 22±1 | 19±1 | 24±2 | 17±3 | 25±3 | 72±6 |
| Cd | NM_PL14_1 | <14 | <60 | <37 | <270 | <900 | <1122 |
|  | NM_PL14_2 | <22 | <125 | <53 | NA | <2337 | <4704 |
|  | NM_PL14_3 | <6 | <24 | <14 | <73 | <330 | <446 |
|  | NM_PL35_1 | <10 | <44 | <27 | <183 | <183 | <509 |
|  | NM_PL35_2 | <7 | <39 | <12 | <70 | <274 | <462 |
|  | NM_PL35_3 | <16 | <66 | <105 | <185 | <787 | <1007 |
|  | M_PL22_1 | 47±6 | <24 | 82±13 | 56±27 | <298 | <431 |
|  | M_PL22_2 | 12±5 | <51 | <24 | <272 | <390 | <1097 |
|  | M_PL22_3 | 53±10 | 66±26 | 55±19 | <269 | <248 | <1658 |
|  | M_PL27_1 | <15 | <76 | <37 | <185 | <272 | 100±99 |
|  | M_PL27_2 | 9±4 | <31 | <29 | <110 | <106 | <730 |
|  | M_PL27_3 | <18 | <83 | <39 | <180 | <813 | <1702 |

**
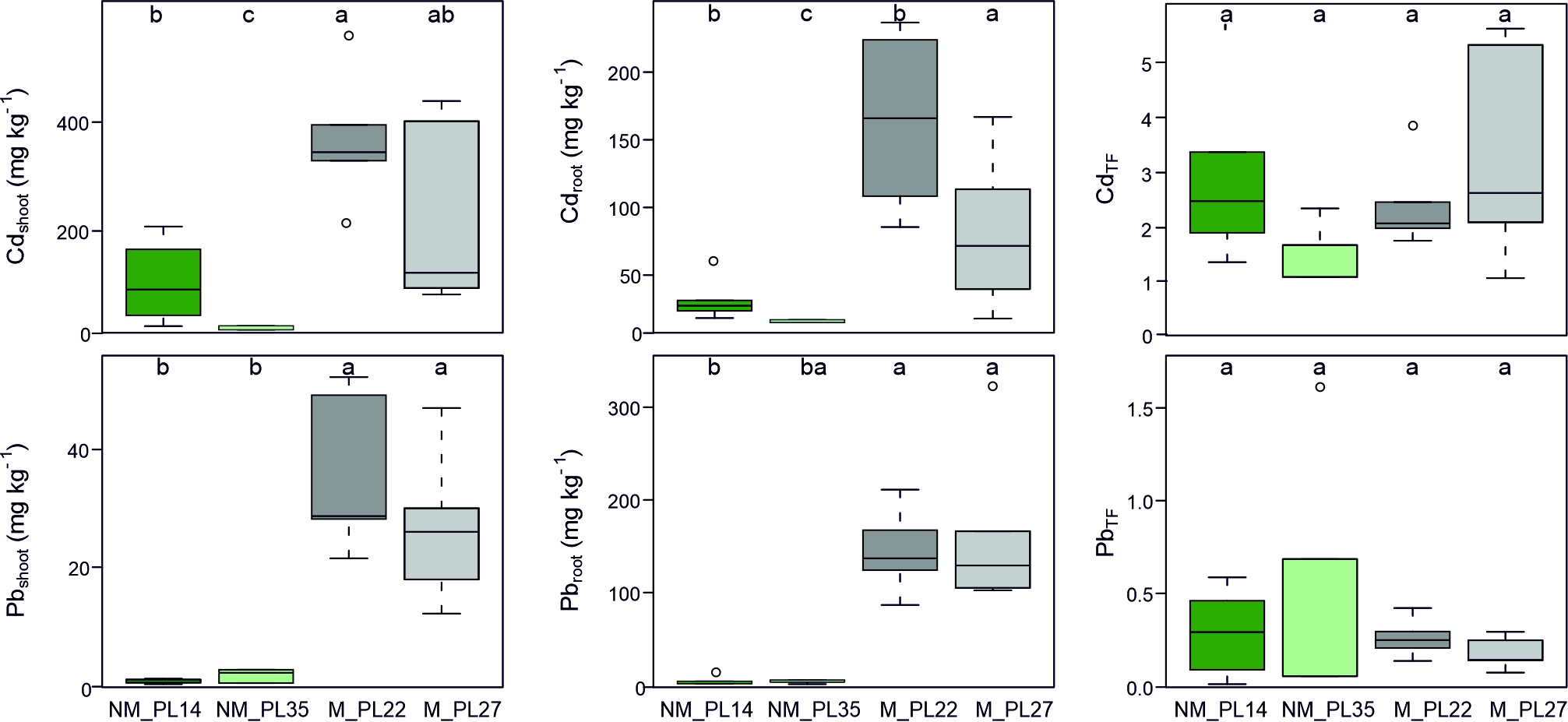
**

**Figure S1** Concentration of Cd and Pb in shoots and roots, and translocation factor (TF) in non-metallicolous (NM) and metallicolous (M) *Arabidopsis halleri* populations. The box represents the 25^th^ and 75^th^ percentiles of the data, the whiskers indicate the first and the fourth quartiles, the median is indicated by the horizontal line. Different letters indicate statistically significant differences at P ≤ 0.05.
